# Improved Medication communication and Patient involvement At Care Transitions (IMPACT-care): study protocol for a pre–post intervention trial in older hospitalised patients

**DOI:** 10.1136/bmjopen-2025-099547

**Published:** 2025-05-02

**Authors:** Henrik Cam, Kristin Franzon, Victoria Östman, Sofia Kälvemark Sporrong, Thomas Gerardus Hendrik Kempen, Elisabet I Nielsen, Karl-Johan Lindner, Beatrice Ekelo, Cecilia Bernsten, Ulf Ehlin, Stina Lindmark, Nermin Hadziosmanovic, Ulrika Gillespie

**Affiliations:** 1 Department of Pharmacy, Uppsala University, Uppsala, Sweden; 2 Department of Public Health and Caring Sciences, Uppsala University, Uppsala, Sweden; 3 Utrecht Institute for Pharmaceutical Sciences, Utrecht University, Utrecht, The Netherlands; 4 Department of Pharmacy, Region Västmanland, Västerås, Västmanland County, Sweden; 5 Östhammar Association of Relatives and Elderly People, Östhammar, Sweden; 6 Geriatrics, Uppsala University Hospital, Uppsala, Sweden; 7 Uppsala Clinical Research Center, Uppsala, Sweden

**Keywords:** Clinical Protocols, Health Services for the Aged, Hospital to Home Transition, Medication Adherence, Patient-Centered Care, Pharmacists

## Abstract

**Introduction:**

Care transitions, particularly hospital discharge, present significant risks to patient safety. Deficient medication-related discharge communication is a major contributor, posing substantial risk of harm to older patients. This protocol outlines the Improved Medication communication and Patient involvement At Care Transitions (IMPACT-care) intervention study, designed to evaluate the effects of a multifaceted intervention for older hospitalised patients on medication-related discharge communication compared with usual hospital care.

**Methods and analysis:**

A pre–post intervention study will be conducted in two surgical and one geriatric ward of a university hospital in Sweden. The study will begin with a control period delivering usual care, followed by a training period and then an intervention period. The intervention comprises four components performed by clinical pharmacists: (1) information package provided to patients and/or informal caregivers, (2) preparation of medication-related discharge documentation, (3) facilitation of discharge communication and (4) follow-up call to patients or their informal caregiver. Eligible participants are aged ≥65 years, manage their own medications independently or with informal caregiver support, and are admitted to the study wards. Each study period (control and intervention) will last until 115 patients have been included. The primary outcome is the quality of medication-related discharge documentation, assessed using the Complete Medication Documentation at Discharge Measure (CMDD-M). Secondary outcomes include patients’ perceptions of knowledge and involvement in discharge medication communication, and their sense of security in managing medication post-discharge; adherence to medication changes from hospitalisation that persist after discharge; and unplanned healthcare visits following discharge. A process evaluation is planned to explore how the intervention was implemented. Patient inclusion began in September 2024.

**Ethics and dissemination:**

The study protocol has been approved by the Swedish Ethical Review Authority (registration no.: 2023-03518-01 and 2024-04079-02). Results will be published in open-access international peer-reviewed journals, and presented at national and international conferences.

**Trial registration number:**

NCT06610214.

**STRENGTHS AND LIMITATIONS OF THIS STUDY**

- Uses a comprehensive, multifaceted intervention designed to address gaps in medication communication both during hospitalisation and after discharge.
- Conducted in both non-surgical and surgical wards, increasing the generalisability of findings to other healthcare settings.
- The inclusion of a process evaluation provides insights into the implementation and adherence to intervention components, offering valuable information to understand and interpret the study findings.
- The pre–post design without randomisation limits the ability to establish causal relationships between intervention and observed outcomes.
- Due to the complex, multifaceted nature of the intervention, it is not possible to determine which specific intervention components contribute most to the observed effects.

## Introduction

 The ageing population is rapidly increasing, with individuals aged 65 and older expected to rise from 10% in 2022 to 16% by 2050.^1^ Older adults frequently experience multiple chronic conditions, making them two times as likely to require hospital care compared with younger adults.^2^ Medications are a primary treatment for many health conditions, and as the prevalence of multiple illnesses increases, so does the use of medications, increasing the risk of medication-related complications.^3 4^ One in six hospital admissions and one in five readmissions among older patients are medication-related,^5 6^ most of which are preventable.^7^ Care transitions, particularly hospital discharges, pose significant risks to patient safety and are highlighted by the World Health Organization as a focus for healthcare improvements.^8^ More than one-third of older patients experience adverse drug reactions within 8 weeks post-discharge,^9^ often attributed to poor communication and coordination between hospitals, subsequent healthcare providers and patients or their informal caregivers.^610^,^13^ Most hospitalised older patients experience changes to their medication regimens, which persist after discharge and should be effectively communicated to all individuals involved in their care.^14 15^

Relying on written discharge notes and referrals to bridge communication gaps regarding medication changes and follow-up plans has proven unreliable, as this information is often delivered late or of insufficient quality.^16^,^19^ Discharge consultations often lack structure and patient-centredness, frequently being treated as a checklist item for healthcare professionals (HCPs) to complete before discharge.^20^,^22^ Physicians tend to adopt an authoritative role in medication discussions, which can discourage older patients from actively participating in their medication management.^23^ To foster patient involvement, HCPs should act as advocates rather than paternalistic figures.^24^ Patient-centred communication at discharge is essential for equipping patients with the knowledge and confidence to manage their medications and self-care.^20^ Involving patients in medical decisions is a key component of patient-centred care, leading to improved patient satisfaction and clinical outcomes, such as better glycaemic and blood pressure control.^25 26^ However, older patients may be less inclined or unable to participate actively, often due to factors such as cognitive or physical impairments.^27^ Many feel insufficiently empowered to engage in discussions about their medications and tend to rely on HCPs, following prescriptions without question.^22 28^ Even when discharge information is presented in a structured format, older patients frequently struggle to retain details about their medications.^29^ Informal caregivers can be vital in supporting patient involvement and bridging communication gaps between HCPs and older patients.^12 23^

To address these issues, the research project Improved Medication communication and Patient involvement At Care Transitions (IMPACT-care) was initiated.^30^ The project began with exploratory studies of the discharge communication,^12 19 22^ ultimately leading to the development of the intervention presented in this protocol.

### Aims and objectives

The overall aim is to evaluate the effects of a multifaceted intervention on improving medication-related discharge communication for older hospitalised patients, compared with usual hospital care.

The primary objective is to assess the intervention’s impact on the quality of written medication-related discharge documentation compared with usual hospital care. Secondary objectives include evaluating the intervention’s effect on patients’ perceived involvement in discharge medication communication and their confidence in post-discharge medication management, as well as adherence to medication changes from hospitalisation that persist after discharge and the need for unplanned healthcare visits following discharge, all in comparison to usual hospital care.

## Methods and analysis

This protocol was developed and reported in accordance with the Standard Protocol Items: Recommendations for Interventional Trials (SPIRIT) 2013 statement,^31^ the SPIRIT-outcomes 2022 extension,^32^ and the Template for Intervention Description and Replication checklist.^33^

### Study design

**Figure 1:**
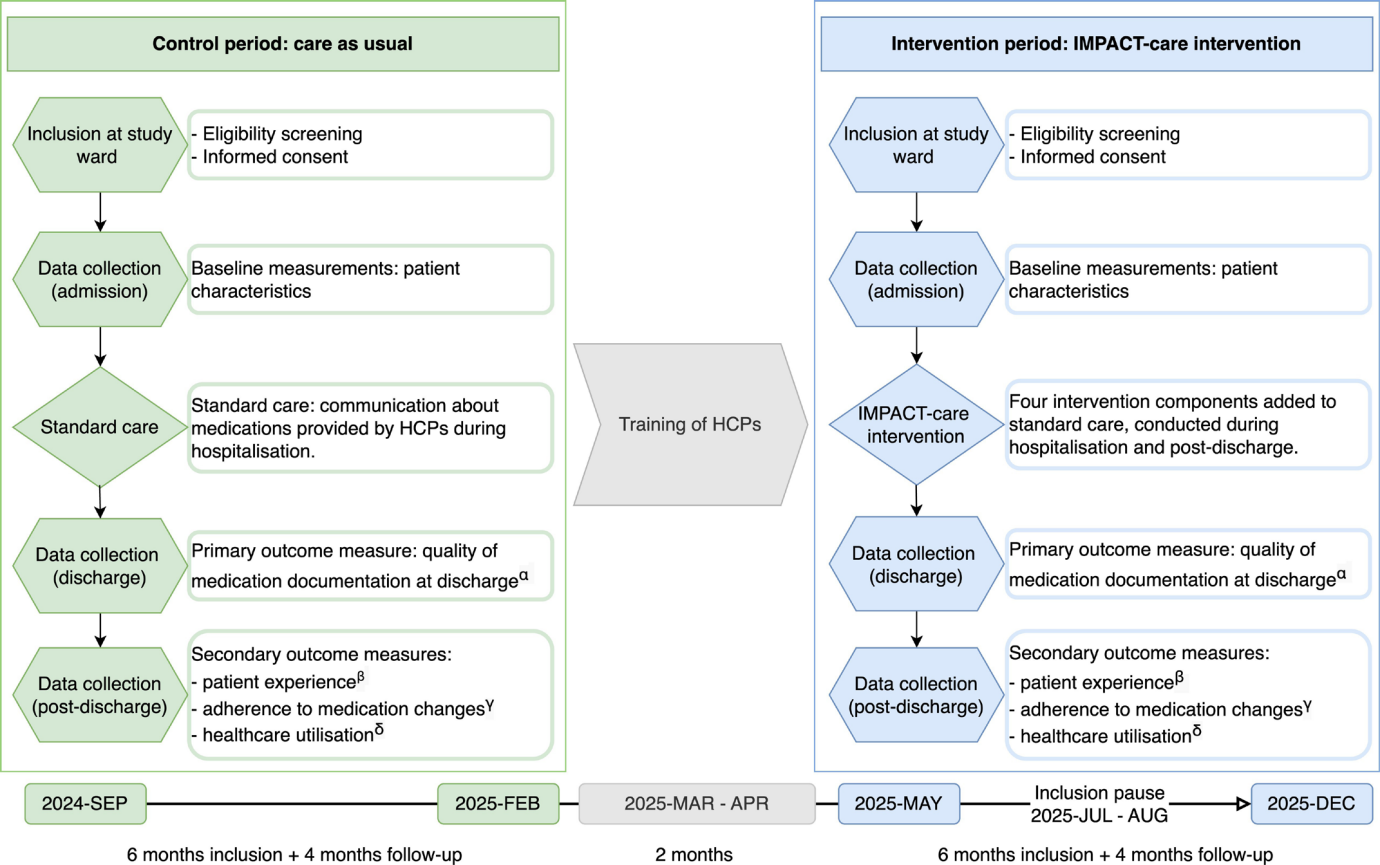
Schematic overview of the study design. ^α^CMDD-M, a point-based instrument using data from the patient’s electronic health records. ^β^PIMCH-Q, a questionnaire to patients measuring their perceptions of involvement in discharge medication communication and their confidence in post-discharge medication management. ^γ^Data on lasting medication changes from the patient’s electronic health records are compared with pharmacy dispensing data collected 120 days post-discharge. ^δ^Unplanned hospital revisits and medication-related readmissions up to 90 days post-discharge. CMDD-M, Complete Medication Documentation at Discharge Measure; HCPs, healthcare professionals; IMPACT-care, Improved Medication communication and Patient involvement At Care Transitions; PIMCH-Q, Patient Involvement in Medication Communication at Hospital discharge Questionnaire.

This prospective intervention study uses a pre–post design (figure 1). Control patients will be enrolled first (control period), followed by a training phase during which HCPs in the study wards will be trained to implement the intervention. Once the HCPs have undergone training sessions, the intervention period will start. Enrolment during both the control and intervention period will stop once the target sample size is reached, with patient follow-up continuing for 4 months post-discharge. Based on the pilot study, the control and intervention periods are each expected to last approximately 6 months, and the training phase will last around 2 months. Figure 1 provides a schematic overview of the study design. Study enrolment began in September 2024, and recruitment of participants for the intervention group is currently ongoing as of April 2025.

#### Rationale for study design

A randomised trial was deemed infeasible—neither at the patient level, due to contamination risks, nor at the ward level or as a stepped-wedge design, as these would require a large number of wards and exceed available resources. Consequently, a pre–post study design was selected, complemented by an interrupted time series (ITS) analysis for exploratory purposes. The ITS analysis, analysing data at regular intervals both before and after the intervention, allows for a more nuanced interpretation of the primary results by accounting for potential seasonal variations and changes in effect over time.

### Settings

The study is conducted in two surgical wards and one geriatric ward at Uppsala University Hospital in Sweden. The surgical wards mainly handle emergency surgeries, as well as liver-pancreas, transplantation, oesophagus-stomach, endocrine and colorectal surgeries. The geriatric ward treats older patients with complex acute medical and rehabilitation needs. These two clinical specialties were selected to assess whether the intervention could have an effect across various clinical settings.

### Study population and recruitment

Patients aged 65 years or older, who manage their own medications either independently or with support from an informal caregiver and are admitted to the study wards, are eligible for inclusion. An informal caregiver is defined as an unpaid individual, often a family member, who assists the patient with daily activities, healthcare communication and medication management. Exclusion criteria apply if patients meet any of the predetermined conditions that would hinder the successful delivery of the intervention or the reliable collection of outcome data (a detailed list is provided in box 1).

Box 1 **The inclusion and exclusion criteria in the study. Patients meeting exclusion criteria 1–9 are excluded at the time of hospital admission, while those meeting exclusion criteria 10–15 are excluded at the time of discharge**

**Inclusion criteria**

1. 65 years or older. 
2. Manages their own medications, either independently or with support from an informal caregiver, prior to inclusion.

**Exclusion criteria**

Checked at hospital admission

1. Registered in a region outside the study hospital (limited data availability).
2. Admitted from a nursing home (no own medication management prior to admission).
3. Unable to receive information or provide consent independently (due to cognitive impairment or unresponsiveness).
4. Already included in the study.
5. Patient delocalised to the study ward with another medical discipline responsible for the patient’s care (formally, no study ward patient).
6. In a late palliative phase prior to inclusion (intervention not suitable).
7. Unable to communicate in Swedish (hindering intervention delivery).
8. Has restricted personal information in the EHR (limited data availability).
9. Admitted for transplantation (intervention not suitable).

Checked at hospital discharge

1. Discharged to a nursing home (intervention not suitable).
2. Patient transitions to late palliative phase during the hospitalisation (intervention not suitable).
3. The patient is transferred to a non-study ward and is discharged from there (hindering intervention delivery).
4. The patient dies during the course of the hospital stay (hindering intervention delivery).
5. No medication changes that last post-discharge during the hospitalisation (intervention not suitable).
6. The duration of stay on the study ward is less than 48 working hours (excluding time from 16:00 before weekends/public holiday to 08:00 the day after a weekend/public holiday) (hindering intervention delivery).

EHR, electronic health records.

The researchers, who are employed by the hospital, screen the admission lists of the study wards daily on weekdays to identify eligible patients, who are then asked for inclusion by the researchers or clinical pharmacists on the ward. Eligibility is primarily determined through the patient’s electronic health records (EHR), with any uncertainties resolved through discussions with HCPs at the study wards. Once identified, patients are informed both verbally and in writing, and written informed consent (online supplemental material I) is requested. Patients meeting exclusion criteria 10–15 in box 1 are excluded at discharge.

During the recruitment of control patients, all patients fulfilling the inclusion and exclusion criteria are invited to participate. During the intervention period, patient inclusion is determined based on the capacity of the pharmacists performing the intervention. The pharmacists’ capacity will be evaluated through regular feedback discussions, ensuring that the inclusion process aligns with their workload. If the number of eligible patients exceeds the pharmacists’ capacity, the pharmacist, in collaboration with the research team, will determine how many eligible patients can be included. To prioritise which patients to include, a random priority number will be generated for each eligible patient at the study ward level using Microsoft Excel, with those assigned the highest priority included first.

### Intervention development

The intervention aims to improve medication communication during the discharge process for older patients. It was designed by a multidisciplinary team comprising researchers with backgrounds in social science, pharmacy, medicine and nursing. Several team members also work professionally as healthcare practitioners in clinical settings, contributing practical insights from ongoing patient care. In addition, the team included two public representatives, ensuring that the perspectives of patients and informal caregivers were meaningfully integrated. The design built on findings from previous research conducted by our group.^12 19 22^ The inclusion rate, as well as the feasibility of selected intervention components and outcome measures, was tested in unpublished pilot studies conducted at geriatric and surgical wards at Uppsala University Hospital, Sweden. These studies involved a total of 106 patients between September 2023 and May 2024 (Nordin J, Berlin K, Sabouni Y, du Thinh C, *et al*: Facilitating patient empowerment at hospital discharge: A pilot study testing the feasibility of the IMPACT-care intervention, unpublished). Based on the results of these pilot studies, the intervention and study design were refined before advancing to the main trial.

### Control period (pre-intervention)

During the control period, care as usual will be provided at the study wards. Clinical pharmacists are part of the care team at the wards and primarily assist with medication reviews at patient admission and discharge but are not routinely involved in the discharge communication process. At hospital admission, medication reconciliation is conducted by either a pharmacist or a physician. If needed, a medication review is carried out by the physician, with or without support from a pharmacist. Any changes to the patients’ medication lists are made by wards physicians or nurse practitioners (specialised nurses at the surgical wards). Oral medication-related communication with the patient and/or informal caregiver is typically handled by nurses, physicians and pharmacists during patient consultations. At discharge, hospitals are required to provide a discharge summary to the next healthcare provider(s) and a discharge letter intended for the patient.^34 35^ Both documents are typically prepared by ward physicians and include details about the hospitalisation, medication changes (along with the rationales for those), planned treatment duration and follow-up plans. The discharge letter, however, is expected to be written in layman’s language. In some cases, these discharge documents are written by a physician who has not met the patient prior to discharge. Pharmacists sporadically assist in preparing these discharge documents, but not in a standardised manner. Additionally, it is standard practice for ward physicians to send specific referrals to the next healthcare provider(s), outlining follow-up requests related to medication changes. In addition, ward physicians conduct an oral discharge consultation, during which the patient is informed about the medication changes and follow-up plans before discharge.

While patients receive written information materials with practical information about the wards and surgical procedures at admission, no materials specifically address medications or medication communication at discharge. Inviting informal caregivers to participate in discharge consultations and HCPs conducting follow-up calls after discharge occurs in selected cases but is not routine practice.

### Implementation period: training of HCPs

The training period will last approximately 2 months between the control and intervention phases. During this period, HCPs—primarily physicians and pharmacists on the study wards—will undergo training. Training of physicians will focus mainly on the importance of writing discharge documentation and effectively using pharmacist support for this process. Training of pharmacists, on the other hand, will focus on implementing the intervention components and understanding the principles of person-centred medication communication at discharge. The training will be delivered through multiple sessions led by the researchers, addressing how the study may impact daily ward processes and how to integrate the intervention components into existing practices. To accommodate newly hired HCPs during the intervention period, as well as those unable to attend the live sessions, digital training materials will be developed and distributed to ensure that all necessary training can be completed. Additionally, the pharmacists, who play a central role in delivering the intervention components, will receive a standardised operation procedure document. Other HCPs on the wards, excluding physicians and pharmacists, will be informed about the study through meetings and information emails, which will outline how they may be affected by the study. For training purposes, selected patients will undergo the intervention components without being included in the study. Additionally, one of the researchers will also regularly visit the study wards to support the HCPs in implementing the intervention components during this phase.

### Intervention period

**Figure 2:**
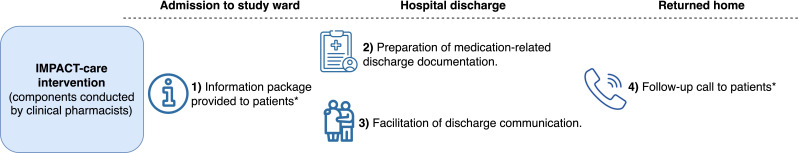
Overview of the IMPACT-care intervention, comprising four intervention components implemented during patient hospitalisation and post-discharge. *Based on the patient’s preference, this may include their informal caregiver. IMPACT-care, Improved Medication communication and Patient involvement At Care Transitions.

The intervention is designed to be implemented on hospital wards by clinical pharmacists who are already part of the patient care team. Each of the study wards in our study has a full-time equivalent clinical pharmacist present during weekday office hours, with a continuous presence established over the past 15 years before the study began. The pharmacists involved have varying levels of experience, from limited to more extensive, some of whom have a 1-year full-time postgraduate MSc in clinical pharmacy. All relevant details about the completed intervention components and any other actions taken by the pharmacist will be documented as usual in the patient’s EHR. The IMPACT-care intervention consists of the following four components (figure 2).

#### Component 1: Information package provided to patients and/or informal caregivers

In our pre-study,^22^ it was identified that medication-related discharge communication is not tailored to support patients’ self-care needs post-discharge. Additionally, patients were unprepared for medication-related consultations prior to discharge. To address these challenges, the research team, inspired by a similar intervention component developed in the UK,^36^ designed an information package consisting of a patient booklet (online supplemental material II) with input from clinical pharmacists at Uppsala university hospital and a panel of public representatives.

The booklet is designed to inform, prepare and engage patients and/or their informal caregiver in medication communication at discharge and self-care after returning home. It is organised into four sections: (1) hospitalisation course, (2) medications, (3) discharge and (4) advice on self-care. The first two sections feature a question prompt list,^37^ a set of discussion points intended to guide conversations with HCPs and encourage patients to actively participate in their care. The use of the questions prompt list has been found to enhance patient participation in medication-related communication.^38 39^ The third section contains a checklist of essential points for patients to review with HCPs to help confirm they have sufficient knowledge before leaving the hospital. The final section provides practical advice on seeking medication and general healthcare support after returning home.

Patients will receive the booklet in printed format at admission to the study ward and will be accompanied by an oral consultation with one of the researchers who also practices clinically as a pharmacist. During the consultation, the pharmacist will explain the content and guide the patient on how to use the booklet effectively. The booklet will also be available online. If the patient wishes, the pharmacist will provide the patient’s informal caregiver access to the information package. This can be done in person if the caregiver is present at the ward or remotely via phone, guiding them on how to access the materials online.

#### Component 2: Preparation of medication-related discharge documentation

Incompleteness and poor quality of medication-related discharge communication from hospitals is a common problem,^40 41^ making it difficult for subsequent HCPs to trust this information.^12 17 42^ Pharmacist involvement can significantly improve the completeness and quality of such communication.^40 41^ Consequently, in our study, the pharmacist will review relevant parts of the patient’s EHR and medication list prior to discharge to identify any lasting medication changes made during the hospitalisation. The pharmacist will collaborate with the discharging physician to reconcile follow-up plans for these changes. All medication changes, including reasons for the adjustments (when known), planned treatment duration, follow-up plans and the ward’s phone number for any post-discharge inquiries from the patient, will be documented in a standardised manner in the EHR by the pharmacist. This documentation will form the basis for detailing medication changes and follow-up plans in the patient’s discharge letter and the discharge summary intended for the next healthcare provider, both of which are written by a ward physician.

#### Component 3: Facilitation of discharge communication

To increase the likelihood that patients and their informal caregiver remember and use the booklet provided to them during intervention component 1, the pharmacist will consult the patient as the discharge date approaches. The consultation will include a review of the booklet’s content and a reminder to use it. If the patient wishes, the pharmacist will also contact the patient’s informal caregiver, either by phone or face-to-face, depending on the situation, to review the booklet and remind them to use it.

Informal caregivers are considered valuable support in helping patients recall information and manage self-care after returning home.^12^ However, they are often involved in a limited way in medication-related discharge communication by HCPs.^22 43^ To address this gap, the pharmacist in our study will arrange for an informal caregiver to attend the discharge consultation with the physician, if the patient so wishes. The pharmacist will contact the informal caregiver by phone once the discharge date is confirmed—no later than the morning of discharge—and invite them to participate in the consultation. Participation can be in person, by phone, or via video call depending on their availability. The pharmacist will then inform the discharging physician that the patient has requested their informal caregiver’s involvement in the discharge consultation.

#### Component 4: Follow-up call to patients or their informal caregiver

The timing of medication-related discharge communication often occurs at a suboptimal moment for patients, making it difficult for them to retain and recall the information after returning home.^22^ Incorporating intervention components both during hospitalisation and after discharge can help support medication continuity in older patients and bridge transitions.^44^ Telephone follow-ups, in particular, have shown promise in enhancing this support.^44^ Therefore, in our study, patients or their informal caregiver (based on the patient’s preference) will be offered a follow-up call with a clinical pharmacist post-discharge. If requested, the appointment for this call will be scheduled in consultation with the patient/informal caregiver, between 3 and 7 days after discharge, depending on the patient’s availability. The pharmacist will then contact the patient/informal caregiver at the agreed time. During the call, the pharmacist will start by addressing any questions the patient/informal caregiver may have, providing direct answers or referring inquiries to the appropriate HCP as needed. Following this, the pharmacist will review the medication-related discharge information, focusing on the updated medication list and details outlined in the discharge letter, including medication changes, reasons for the adjustments, planned treatment duration and follow-up plans. Additionally, the pharmacist will remind the patient of the advice on when and how to seek care, as presented in the booklet provided during intervention component 1.

### Outcomes

All outcomes will be assessed for participants from both the control and the intervention group (table 1).

**Table 1:** Timeline and overview of the scheduled data collection for both the control and intervention group participants

	Admission	Discharge	Follow-up				
Time points (day)	−1*	0	7	14	30	90	120
Study inclusion							
Eligibility screening	x	x					
Informed consent	x						
Demographic data	x	x					
Outcome measures							
CMDD-M†		x					
PIMCH-Q‡			x				
Adherence to medication changes				x			x
Hospital revisits			x		x	x	
Hospital readmissions			x		x	x	
Emergency department visits			x		x	x	
Medication-related readmissions			x		x	x	

* Time point at which the patient is admitted to the study ward.

† CMDD-M, a point-based instrument using data from the patient’s electronic health records.

‡ PIMCH-Q, a questionnaire to patients measuring their perceptions of involvement in discharge medication communication and their confidence in post-discharge medication management.

CMDD-M, Complete Medication Documentation at Discharge Measure; PIMCH-Q, Patient Involvement in Medication Communication at Hospital Discharge Questionnaire.

#### Primary outcome

The improvement in the quality of medication-related discharge documentation will be the primary outcome, assessed using the average score from the Complete Medication Documentation at Discharge Measure (CMDD-M) (online supplemental material III).^45^ This point-based instrument, ranging from 0 to 9 points, is based on Swedish legislation^35^ outlining the requirements for written medication-related discharge documentation. The CMDD-M comprises five items, each scored from 0 to 1 or 0–2 points depending on the criteria. It evaluates the completeness and quality of medication-related discharge documents for individual hospital discharges, including the patient’s discharge letter, the discharge summary intended for the next healthcare provider and the presence of a follow-up request to bridge the gaps post-discharge. Improving the quality of discharge documentation is critical for ensuring continuity of care and patient safety during transitions of care.^17^ Poor-quality documentation has been associated with medication errors,^11^ non-adherence^46^ and avoidable need for medical care after discharge.^47 48^ By focusing the primary outcome on this domain, the trial aims to address an important gap in care that impacts patient outcomes.

#### Secondary outcomes

- The proportion of patients with complete medication-related discharge documentation. This will be assessed using the CMDD-M to determine the prevalence of patients achieving the maximum score of 9 points.
- Improvement in patients’ perceptions of knowledge and involvement in discharge medication communication, and their sense of security in post-discharge medication management. This will be assessed by the average score by the Patient Involvement in Medication Communication at Hospital discharge Questionnaire (PIMCH-Q) (online supplemental material IV). It consists of eight statements rated on a 4-point Likert scale. It is designed with three dimensions: perception of knowledge, involvement and sense of security and aims to measure patients’ perceived involvement in medication communication during hospitalisation and their sense of security in managing medications after discharge. The questionnaire will be sent to patients 1-week post-discharge.
- Adherence to medication changes made during hospitalisation that persist post-discharge. This will be assessed by measuring the number of instances of non-adherence. Non-adherence to a medication change is defined as follows:
  1. New or modified medications: a medication initiated or modified during hospitalisation (ie, changes in strength, dose or formulation) that is not dispensed from a community pharmacy within 14 days post-discharge. This applies regardless of whether the reason is a missing prescription or the patient not filling the prescription. Changes in strength or dose are included only when they create a safety risk for the patient if the previous prescription is used (eg, reducing the dose of a tablet from 10 mg once daily to 2.5 mg once daily, which would require the patient to split the same tablet two times to get the correct dose, a practice considered unsafe).
  2. Discontinued medications: a medication discontinued during hospitalisation (or with altered strength or formulation), that is, erroneously dispensed using a previous prescription within 120 days post-discharge.
    The rationale for collecting data up to 120 days post-discharge is based on standard pharmacy practices in Sweden, where medications are typically dispensed for a 90-day (3-month) supply at a time. Patients may have leftover supplies of discontinued medications at home and continue using them. However, if these medications are not refilled at a pharmacy within 120 days, the patient is considered adherent to the discontinuation.
- The proportion of patients who are fully adherent to the medication changes made during hospitalisation that persist post-discharge. This will be assessed by determining the prevalence of patients who have no instances of non-adherence as described above.
- Unplanned healthcare visits post-discharge. This will be assessed using the following outcome measures:
  - The prevalence of patients with at least one unplanned hospital revisit (a composite measure of unplanned readmissions and emergency department visits) at 7, 30 and 90 days post-discharge.
  - The prevalence of patients with at least one unplanned readmission at 7, 30 and 90 days post-discharge.
  - The prevalence of patients with at least one emergency department visit (not followed by admission) at 7, 30 and 90 days post-discharge.
  - The time to the first unplanned hospital revisit within 90 days.
  - The time to the first unplanned readmission within 90 days.
  - The time to the first emergency department visit within 90 days.
  - The prevalence of patients with at least one potentially medication-related hospital readmission at 7, 30 and 90 days post-discharge, assessed using the validated Assessment Tool to identify Hospital Admissions Related to Medications (AT-HARM10).^49 50^

### Data collection

Screening of patients at the study wards will be performed by the researchers, who are employed by the hospital. This will be done using information from the EHR and, if any unclarities occur, through contact with the ward HCPs. The researchers will invite eligible patients to participate, and patients willing to participate will be asked to sign informed consent (online supplemental material I). Data will be collected from all participants, regardless of their adherence to the intervention, provided they do not withdraw their consent to participate in the study. This approach ensures complete follow-up data for inclusion in the intention-to-treat (ITT) analysis. The data collection will proceed in several steps (table 1) and will be conducted by researchers in the research team and trained research assistants. To ensure uniformity of data collection, standard operating procedures have been developed. Data will be pseudonymised and transferred to case report forms (CRFs) in an electronic data capture system, Research Electronic Data Capture (REDCap).^51^ All data processing and analysis will be based on the data in these CRFs and will be shared and discussed in pseudonymised form. Any forms and electronic files that reveal research data of an individual patient will be stored in a locked archive at the hospital pharmacy. Access to the final trial data set will be restricted to the members of the research team.

#### Demographic data

Demographic data collected from the EHR will include age, gender, renal function, admission and discharge dates, medication treatment at admission and discharge, whether the patient has support by automatic dose-dispensation of medications, disease diagnoses, primary diagnosis for admission, home care support, whether the patient lives alone and the number of emergency department visits and hospital admissions in the past year. Information about patients’ education level will be gathered through the researchers asking the patients at inclusion.

#### Completeness and quality of discharge documentation

After patient discharge, the discharge letter, discharge summary and referrals to next healthcare providers for follow-up will be extracted from the EHR for scoring according to CMDD-M (online supplemental material III). The instrument was specifically developed to be used in clinical settings in Sweden. Initial validation demonstrated that the instrument is feasible for use in our setting.^45^ Inter-rater reliability was assessed using Cohen’s weighted kappa with both linear (Kw linear) and quadratic (Kw quadratic) weights. The Kw linear for the comparison between two clinical pharmacists was 0.92, while the comparison between their consensus and a geriatrician yielded a Kw linear of 0.64. Similarly, the Kw quadratic was 0.97 for the comparison between the pharmacists and 0.80 for the comparison between their consensus and the geriatrician. These findings indicate moderate to almost perfect reliability between raters and suggest that the CMDD-M instrument provides robust reliability in assessing the quality and completeness of medication-related discharge documentation in older hospitalised patients.^45^ The CMDD-M was selected as the primary outcome, as it was deemed the most appropriate and feasible option. Although only component 2 (preparation of medication-related discharge documentation) of the intervention is expected to have a direct effect on this measure, components 1 (information package provided to patients) and 3 (facilitation of discharge communication) are also expected to exert indirect effects on the CMDD-M score by encouraging patients/informal caregivers to request the discharge documents to which they are entitled and to ask more questions about their medications. This, in turn, is anticipated to prompt physicians to provide more explicit information in the documentation. Several alternative outcomes were considered but found to be less suitable, for example, unplanned hospital revisits would require an unfeasibly large sample size; the PIMCH-Q lacks complete validation; and measuring adherence to medication changes raised concerns about precision. These outcomes were therefore designated as secondary. Given its design and focus on aspects directly relevant to our intervention, we anticipate it to effectively capture meaningful changes within our study sample, even though the responsiveness of the CMDD-M to changes in the completeness and quality of discharge documentation in response to an intervention has not yet been evaluated. To ensure objectivity, the assessment using the CMDD-M will be conducted by the researchers in a blinded manner. Data extracted from the EHR will be masked to prevent assessors from linking patients to specific time periods, ensuring they remain unaware whether the patient belongs to the control or intervention group.

#### Patients’ experience

The PIMCH-Q will be sent to patients by mail or email, depending on their preferences, 1 week after the discharge date (online supplemental material IV). The patients are asked to answer the questionnaire as soon as possible. If no response is received within 10 days, the research team will follow up with a reminder via email or phone. During the reminder call, patients will also be offered the option to respond by phone if preferred. The PIMCH-Q was selected for this study because, to the best of our knowledge, no existing instrument adequately captures medication-related patient experiences during hospital discharge. While its responsiveness has not yet been validated, the tool was specifically designed to assess patient involvement in medication communication and confidence in medication management post-discharge, which are core aspects of this study. Despite the need for further validation, the PIMCH-Q remains the most suitable tool for achieving our study objectives.

#### Adherence to medication changes

Data about the lasting medication changes made and prescribed during hospitalisation will be gathered from the EHR. Information on medications dispensed from pharmacies for each patient 120 days post-discharge will be obtained from the Swedish National Board of Health and Welfare’s national prescribed drug register. This register contains data on all medications dispensed from community pharmacies in Sweden on a patient level. The extracted data will include the medication name, the anatomical therapeutic chemical code, strength, prescribed quantity, collected quantity, prescription date, collection date, prescriber’s profession and workplace. The assessment of the number of instances of non-adherence will be conducted by the researchers.

#### Healthcare utilisation

Unplanned hospital revisits, readmissions, emergency department visits and time to these hospital revisits within 90 days will be extracted from the EHR. The assessment of whether the hospital readmissions were potentially medication-related will be conducted retrospectively and blinded, using the AT-HARM10 tool^49^ through information from the EHR. The assessment will be conducted by one clinical pharmacist and one physician who are not otherwise involved in the study. Initially, they will independently evaluate each case, followed by a discussion to reach consensus on cases where their initial assessment (eg, whether a readmission is potentially medication-related) differed.

### Process evaluation

A mixed-method approach, combining both quantitative and qualitative methods, will be used for a process evaluation to assess adherence to the study protocol and explore the implementation of the intervention. The evaluation will be guided by the framework for process evaluation developed by the United Kingdom Medical Research Council.^52^

#### Quantitative process evaluation

The quantitative process evaluation will include all patients in the study to gain insight into the extent of intervention implementation, the degree to which some intervention components may already be in place during the control period and adherence to the study protocol. The following data will be collected from the EHR:

- The proportion of control and intervention patients who received a discharge letter.
- The proportion of control and intervention patients for whom the clinical pharmacist prepared medication discharge documentation.
- The proportion of intervention patients who received the information package.
- The proportion of control and intervention patients for whom the physician used the medication discharge documentation prepared by the pharmacist. This is measured by manually comparing the content of the prepared medication discharge documentation by the pharmacist with the actual medication summary in the discharge letter and final note written by the physician.
- The proportion of intervention patients who are reminded by the pharmacist to review the information package.
- The proportion of intervention patients who wish to have an informal caregiver present at the discharge consultation, and the proportion of those cases where the pharmacists contacts the informal caregiver to be present.
- The proportion of intervention patients who wish to have a follow-up call with a pharmacist after discharge, received the follow-up call and whether it led to any pharmacist intervention, including details of the intervention.

Additional data collection methods:

- The proportion of all employed physicians and clinical pharmacists at the study wards who attend the training sessions. All HCPs attending the training sessions will be registered by the researchers. Data on HCPs who complete digital training sessions will be extracted from the digital training platform.
- The response rate of PIMCH-Q, along with the distribution method (paper, telephone or digital). This will be extracted from REDCap.
- The proportion of control and intervention patients who recall having a discharge consultation, whether they wished to have an informal caregiver present, whether an informal caregiver was actually present, their desire for a follow-up call and whether they received one. For control patients, these questions aim to determine the extent to which intervention components are performed as part of standard care. Additionally, for intervention patients, the proportion of patients who recall receiving the information package (intervention component 1) and their perception of it will be asked. These questions will be sent to patients alongside the PIMCH-Q.
- The amount of time used by pharmacists to deliver each component of the intervention, as well as the overall intervention, will be measured in a subset of the sample.

#### Qualitative process evaluation

Regular meetings with the pharmacists delivering the intervention will be scheduled during the intervention period to discuss and address implementation barriers that could be resolved to support successful implementation.

At the conclusion of the intervention phase, a qualitative process evaluation will be conducted with HCPs and patients. This will involve semi-structured interviews and focus groups with HCPs, specifically physicians and pharmacists from the study wards, who were actively involved in delivering the intervention, to explore their experiences and perceptions of its implementation. Semi-structured interviews will also be conducted with patients, and when applicable their informal caregiver, who received components of the intervention. This qualitative component will offer insights into how patients perceived the intervention. Patient interviews will be conducted either shortly before or within 1 week after discharge. A purposeful sampling approach will be adopted to obtain maximum variation. For HCP focus groups and interviews, variation will be sought in terms of sex, working experience and study ward.^53^ The same approach will be applied for patient interviews, but this time to capture heterogeneity in age, sex and health complexity. The concept of information power will guide the decision of sample size.^54^ All interviews and focus groups will be audio-recorded and transcribed verbatim. The data will be analysed thematically.^55^

### Sample size calculation

The sample size calculation is based on the primary outcome, which is the quality of medication-related discharge documentation measured using the CMDD-M. The intervention will be deemed successful if the average score is significantly higher in the intervention group compared with the control group. For the calculation, we assumed an evenly distributed sample between the two groups and set the target difference in CMDD-M scores at 1 point. This conservative target was chosen as it represents the smallest measurable step in the instrument. In practice, a 1-point difference may indicate the inclusion of medication changes in the discharge letter or discharge summary. Such an improvement reflects a critical enhancement in quality, with important implications for patient safety and continuity of care. Data from the pilot studies indicated that the baseline value for CMDD-M was 3.9 (SD 2.6) (Nordin J, Berlin K, Sabouni Y, du Thinh C, *et al*: Facilitating patient empowerment at hospital discharge: A pilot study testing the feasibility of the IMPACT-care intervention, unpublished). Due to the maximum score limit in CMDD-M, the variance in scores is expected to differ between the control and intervention periods. This difference arises as scores may cluster near the upper limit, particularly in the intervention period where improved performance is anticipated, potentially leading to reduced variability compared with the control period. A two-sided t-test with Welch’s correction for df (to account for the variance difference between groups) was used. A power of 0.8 was considered sufficient to detect an increase, with a 5% two-sided significance level. Based on these assumptions, a sample size of 115 patients per group, for a total of 230 patients, is required.

Additionally, a permutation test using the Mann-Whitney U test was performed to assess the robustness of the t-test, yielding similar results.

### Statistical analysis

A full statistical analysis plan will be finalised prior to any analyses. Statisticians from the Uppsala Clinical Research Center will oversee the statistical analyses. The primary analysis will follow the ITT principle, including all included patients in their assigned groups, regardless of protocol adherence. Additional analyses will include per-protocol analyses, that is, excluding patients with protocol violations.

Descriptive analyses of the study population will be performed, with continuous data presented as mean±SD for normally distributed variables or as median and range for non-normally distributed variables. Categorical variables will be reported as frequencies and percentages. All outcomes will be summarised by study group, overall and by ward, descriptively. Comparative statistics between study groups will be conducted, with all statistical tests being two-sided and a p value<0.05 considered statistically significant.

Models for analysing primary and secondary outcomes will include both unadjusted and fully adjusted analyses. Adjustments will account for age, gender, education level, ward type (geriatric or surgical), number of medication changes persisting post-discharge, number of medications at discharge, support by automatic dose-dispensation of medications and duration of hospitalisation. Effect estimates, including ORs, HRs and rate ratios will be presented with 95% CI and p values.

#### Primary outcome analysis

Linear regression models with robust SEs will be used to estimate the effect of the treatment groups on the CMDD-M score. The results will be reported as effect estimates. A sensitivity analysis of the primary outcome will be performed using a permutation-based Wilcoxon non-parametric test.

#### Secondary outcome analysis

Logistic regression will be used to analyse the prevalence of patients achieving the maximum score (9 points) on the CMDD-M, with results presented as ORs. The PIMCH-Q score will be analysed using linear regression models, evaluating the three dimensions both separately and in total. Differences in the number of instances of non-adherence to medication changes persisting post-discharge will be assessed using quasi-Poisson regression models, with results reported as rate ratios. Logistic regression models will be used to analyse the prevalence of patients who are fully adherent to medication changes persisting post-discharge, with results reported as OR.

The difference in the prevalence of patients with unplanned hospital revisits, unplanned readmissions, emergency department visits and medication-related readmissions at 7, 30 and 90 days post-discharge will be compared with logistic regression models, with results presented as ORs. Time to first unplanned hospital revisit, time to first unplanned readmission and time to first emergency department visit will be analysed using Cox proportional hazards models. Patients who do not experience the event by the end of the study period or are lost to follow-up will be censored at their last known follow-up time, while patients who die before experiencing the event will be censored at the time of death. Results will be reported as HRs.

#### Exploratory analyses

To analyse data collected at multiple regular intervals before and after the intervention, an ITS analysis will be performed. A linear regression model will be estimated as follows:


$$\begin{array}{l} Y=b_{0}+b_{1}T+b_{2}I+e \end{array}$$

where:

Y: outcome variable (CMDD-M score, prevalence of patients achieving the maximum score on CMDD-M, PIMCH-Q score or the number of non-adherence instances to medication changes persisting post-discharge)

b_0_: intercept, representing the expected value of the outcome variable (Y) at baseline (T=0 and I=0).

b_1_: time effect, indicating the change of the outcome variable (Y) for each day passed, regardless of the intervention.

T: time in days passed from the start of the study, capturing natural changes in the outcome over time.

b_2_: intervention effect, representing the difference in the outcome variable (Y) between pre-intervention (I=0) and post-intervention (I=1) periods, after accounting for time trends.

I: dummy variable indicating whether the observation was collected before (0) or after (1) intervention, enabling comparison outcomes before and after the intervention.

e: error term, capturing random noise or unexplained variation in the outcome variable (Y).

This model will allow us to investigate whether there is an immediate effect following the intervention. Results will be presented as regression estimates with 95% CI and p values. This analysis will be conducted for the following outcomes: CMDD-M score, prevalence of patients achieving the maximum score on CMDD-M, PIMCH-Q score (the three dimensions separately and total score), the number of non-adherence instances to medication changes persisting post-discharge and prevalence of patients who are fully adherent to medication changes.

#### Process evaluation

Quantitative data from the process evaluation will be presented with descriptive statistics by study group and in total. No formal statistical tests will be performed.

### Public and patient involvement

Two public representatives were involved in our research team throughout the intervention development process: CB, who holds political duties advocating for patients, and UE, who serves as the chairperson of an association for relatives of older patients. Both actively contributed to the design and development of the intervention by attending research meetings and participating in decision-making. Additionally, we engaged an advisory board comprising five public representatives, all of whom are either members of senior associations or have experience as patients receiving hospital care. This panel reviewed and provided suggestions to improve the wording of the consent form for study inclusion and the PIMCH-Q sent to patients. They also played a key role in developing the information package for intervention component 1, offering feedback on its design and content.

## Ethics and dissemination

This study involves human subjects and the handling of sensitive personal health data. Although there is a risk associated with collecting sensitive patient data, we will minimise these risks by adhering to the General Data Protection Regulation^56^ and the Declaration of Helsinki.^57^ All participants will provide written informed consent before participation (online supplemental file 1). The study has been approved by the Ethical Review Authority in Sweden (registration no. 2023-03518-01 and 2024-04079-02).

The aim of this intervention study is to evaluate whether a novel approach to medication-related discharge communication can improve patient care. The comparator chosen for this study is the current standard discharge process (care as usual), selected because it reflects the routine practices patients experience in the study settings and provides a relevant baseline for evaluating the intervention’s impact. During the intervention period, in addition to the usual care, the intervention focuses on enhancing the quality of medication-related communication at discharge, involving patients and/or caregivers in discussions with HCPs and offering a follow-up call after discharge to reinforce information retention. During the clinical pharmacists’ follow-up phone calls with patients in the intervention group, new issues may be identified that need attention. If the pharmacist making the call is not the appropriate person to handle these issues, they will consult with another suitable HCP to ensure the problem is addressed.

We plan to publish the results of the main trial and any substudies in international peer-reviewed open-access journals, as well as present them at national and international conferences. The trial is expected to result in multiple published manuscripts, contribute to at least one PhD thesis and support improved implementation of current Swedish regulations for medication-related discharge communication.^35^

## Supplementary material

10.1136/bmjopen-2025-099547 online supplemental file 1

10.1136/bmjopen-2025-099547 online supplemental file 2

10.1136/bmjopen-2025-099547 online supplemental file 3

10.1136/bmjopen-2025-099547 online supplemental file 4
