# Supplementary material for: Improved Medication communication and Patient involvement At Care Transitions (IMPACT-care): study protocol for a pre–post intervention trial in older hospitalised patients
Source: BMJ Open. 2025 May 2;15(5):e099547. doi: 10.1136/bmjopen-2025-099547 (PMC12049937; doi:10.1136/bmjopen-2025-099547)
Supplement: online supplemental file 1 [file bmjopen-15-5-s001.pdf]

(Unofficial translation from documents in Swedish)

Consent form for patients in the control group

Information for research participants (patients) regarding participation in a research project in Region Uppsala

We would like to ask whether you are willing to participate in a research project. This document provides information about the project and what participation entails.

What is the project about, and why am I being asked to participate?

The aim of the project is to improve the quality of, and patient involvement in, medication communication when patients are discharged from hospital. The goal is to increase patients' sense of security and adherence to their medication treatment, as well as reduce the need for unplanned healthcare visits. We are focusing on patients aged 65 years or older, as many in this group take several medications. The project is carried out on the ward where you are currently receiving care, which is why you are being invited to participate.

The research sponsor for the project is Region Uppsala. The sponsor is the organisation responsible for the project. The study has been approved by the Swedish Ethical Review Authority (approval number: 2023-03518-01).

What does participation in the project involve?

The project consists of a control period, where care is provided according to standard routine, followed by an intervention period where additional measures are implemented to improve the quality of and patient involvement in medication communication. You are being invited to participate in the control group, which means you will receive care according to standard routines and be asked to complete a questionnaire approximately one week after discharge from hospital. It focuses on the medication information you received, and how involved and secure you felt after returning home. It takes approximately 5–10 minutes to complete and can be sent to you by post or email, depending on your preference.

What will happen with my data?

The project will collect and register information about your medication treatment, medications collected from the pharmacy, and any need for healthcare after discharge. This data will be obtained from your electronic health record and the Swedish National Board of Health and Welfare’s medication register, and collection will continue for four months after discharge. Your questionnaire responses will also be recorded. Your name will be replaced with a code, and only researchers involved in the project will have access to the code key (the code linked to your personal identity number), which will be stored in a password-protected digital file within Region Uppsala’s secure network. All data processing and presentation of results will be handled by the research team and Uppsala University in a

way that prevents identification of individual participants. For more information, please see the subheading *Handling of Personal Data* below.

Your participation is voluntary, and you may withdraw at any time without providing a reason. Choosing not to participate or withdrawing will not affect your future care or treatment. If you wish to withdraw, please contact the principal investigator (see below).

Possible risks and consequences of participation

You are covered by the patient insurance system, as with any healthcare service. We assess the risks of participating in this study to be low. Some participants may feel their privacy is affected, since information will be retrieved from electronic medical records and the prescribed drug register. Completing a questionnaire shortly after discharge may also feel burdensome.

How will I be informed of the results?

The study results will be published. If you wish, you may access the results once they are published in a scientific journal and in a more popular science format. You can request the results by contacting the principal investigator.

Principal investigator contact

Ulrika Gillespie, *address*, Phone: xxx, Email: xxx

Handling of personal data

All data will be handled in accordance with applicable confidentiality laws. Your information and responses will be stored in a database in a way that prevents unauthorised access. All physical data generated during the study will be stored for 10 years in a locked cabinet at Region Uppsala.

The data controller is Region Uppsala. Under the EU General Data Protection Regulation (GDPR), you have the right to access your data, correct any errors, request deletion, or restrict how your data is processed. To do so, contact Ulrika Gillespie (details above). The data protection officer can be reached at xxx or xxx. If you are dissatisfied with how your data is handled, you have the right to file a complaint with the Swedish Authority for Privacy Protection.

Consent to participate in the project

I have received verbal and/or written information about the study and have had the opportunity to ask questions. I will retain the written information.

I consent to participate in the project IMPACT-care: Improved medication communication and patient involvement at care transitions

☐ I would like to complete the questionnaires digitally.

Email: ____

Place & date	Signature
	Name
	Personal identity number

Consent form for patients in the intervention group

Information for research participants (patients) regarding participation in a research project in Region Uppsala

We would like to ask whether you are willing to participate in a research project. This document provides information about the project and what participation entails.

What is the project about, and why am I being asked to participate?

The aim of the project is to improve the quality of, and patient involvement in, medication communication when patients are discharged from hospital. The goal is to increase patients' sense of security and adherence to their medication treatment, as well as reduce the need for unplanned healthcare visits. We are focusing on patients aged 65 years or older, as many in this group take several medications. The project is carried out on the ward where you are currently receiving care, which is why you are being invited to participate.

The research sponsor for the project is Region Uppsala. The sponsor is the organisation responsible for the project. The study has been approved by the Swedish Ethical Review Authority (approval number: 2023-03518-01).

What does participation in the project involve?

The project consists of a control period, where care is provided according to standard routine, followed by an intervention period where additional measures are implemented to improve the quality of and patient involvement in medication communication. You are being asked to participate in the latter phase of the project, during which the following elements will be added:

1. A clinical pharmacist on your ward will review your medications before you leave the hospital. The aim is to summarise changes made to your medication treatment during your hospital stay. This information will be documented in your medical record and support the physician responsible for providing written information (the medication list and discharge summary), as per routine, to you and your next healthcare provider before discharge.
2. You and your informal caregiver (if applicable) will receive an information brochure and access to an informational video while you are on the ward. These are designed to help you prepare for discharge and include tips on what to discuss with healthcare staff.
3. If you have an informal caregiver you would like to be informed about your medication changes, the pharmacist can help make arrangements for them to participate (in person or by phone/video call) in the conversation with the physician before you leave the hospital.
4. Before discharge, you will be offered a follow-up phone call with a pharmacist from the ward. This call will be scheduled for approximately one week after discharge. The purpose is to go through the written information (the discharge summary) you received and clarify any uncertainties.
5. About one week after your discharge, you will be asked to complete a questionnaire. It focuses on the medication information you received, and how involved and secure

you felt after returning home. It takes approximately 5–10 minutes to complete and can be sent to you by post or email, depending on your preference.

What will happen with my data?

The project will collect and register information about your medication treatment, medications collected from the pharmacy, and any need for healthcare after discharge. This data will be obtained from your electronic health record and the Swedish National Board of Health and Welfare's medication register, and collection will continue for four months after discharge. Your questionnaire responses will also be recorded. Your name will be replaced with a code, and only researchers involved in the project will have access to the code key (the code linked to your personal identity number), which will be stored in a password-protected digital file within Region Uppsala's secure network. All data processing and presentation of results will be handled by the research team and Uppsala University in a way that prevents identification of individual participants. For more information, please see the subheading *Handling of Personal Data* below.

Your participation is voluntary, and you may withdraw at any time without providing a reason. Choosing not to participate or withdrawing will not affect your future care or treatment. If you wish to withdraw, please contact the principal investigator (see below).

Possible risks and consequences of participation

You are covered by the patient insurance system, as with any healthcare service. We assess the risks of participating in this study to be low. Some participants may feel their privacy is affected, since information will be retrieved from electronic medical records and the prescribed drug register. Completing a questionnaire shortly after discharge may also feel burdensome.

A possible benefit is that we might identify issues that can be communicated to the hospital physician. Additionally, receiving a follow-up phone call one week after discharge may be helpful, as previous studies show that questions often arise after leaving hospital. These questions can be addressed directly during the call or forwarded to the responsible physician.

How will I be informed of the results?

The study results will be published. If you wish, you may access the results once they are published in a scientific journal and in a more popular science format. You can request the results by contacting the principal investigator.

Principal investigator contact

Ulrika Gillespie, *address*, Phone: xxx, Email: xxx

Handling of personal data

All data will be handled in accordance with applicable confidentiality laws. Your information and responses will be stored in a database in a way that prevents unauthorised access. All physical data generated during the study will be stored for 10 years in a locked cabinet at Region Uppsala.

The data controller is Region Uppsala. Under the EU General Data Protection Regulation (GDPR), you have the right to access your data, correct any errors, request deletion, or restrict how your data is processed. To do so, contact Ulrika Gillespie (details above). The data protection officer can be reached at xxx or xxx. If you are dissatisfied with how your data is handled, you have the right to file a complaint with the Swedish Authority for Privacy Protection.

Consent to participate in the project

I have received verbal and/or written information about the study and have had the opportunity to ask questions. I will retain the written information.

I consent to participate in the project IMPACT-care: Improved medication communication and patient involvement at care transitions

☐ I would like to complete the questionnaires digitally.

Email: ____

Place & date	Signature
	Name
	Personal identity number
