# Supplementary material for: Improved Medication communication and Patient involvement At Care Transitions (IMPACT-care): study protocol for a pre–post intervention trial in older hospitalised patients
Source: BMJ Open. 2025 May 2;15(5):e099547. doi: 10.1136/bmjopen-2025-099547 (PMC12049937; doi:10.1136/bmjopen-2025-099547)
Supplement: online supplemental file 2 [file bmjopen-15-5-s002.pdf]

Unofficial translation from document in Swedish

Your medications: from hospital to home

A support guide during your hospital stay to help you prepare for going home

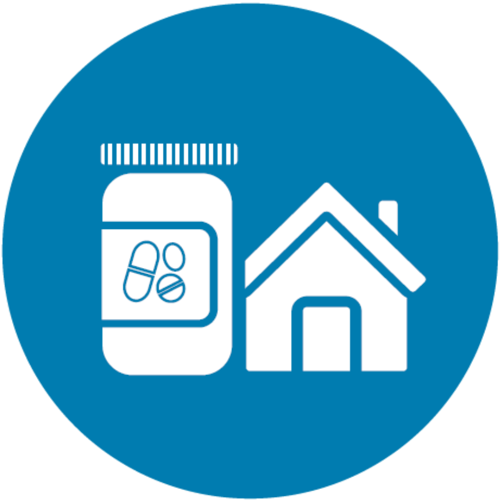

IMPACT-care

How to use this brochure

When staying in a hospital, many things happen, and it may be difficult to understand or feel comfortable with everything that takes place.

This information brochure is designed to support you and your relatives in gaining more knowledge about your care during your hospital stay and after you return home.

It is divided into four sections and includes information and suggested points to discuss with healthcare staff or your relatives.

You can mark the points you would like to discuss with the healthcare staff. You can also write down your own questions.

1 My hospital stay

2 My medications

3 Preparing to go home (discharge)

4 Advice for when I am home

Scan with a mobile camera for an informative film

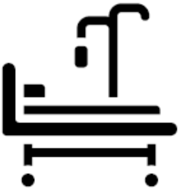

My hospital stay

To be able to take care of your own health and manage your medication treatment when you return home, it helps if you have knowledge of what has happened during your hospital stay.

You can get this knowledge by:

- Talking to the healthcare staff about what is happening and asking if there is anything you are unsure about.
- Asking to be included in discussions about your care and medication treatment.

Points I would like to discuss

Mark the points you would like to discuss and/or write down other questions.

- What have I been treated for during my hospital stay?
- What is the plan for my care while I am still in the hospital?
- What is the plan for my care after I leave the hospital?
- What kind of help and support can I receive when I get home?
- How can my relatives help me?

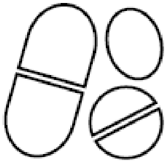

My medications

It is common for medication changes to occur during a hospital stay, and it can be challenging to keep track of them.

You can ask healthcare staff about your medications. You can also discuss how to take them at home and whether you need any practice.

Points I would like to discuss

Mark the points you would like to discuss and/or write down other questions.

- What medications am I taking and why?
- How and when should I take my medications, and are there any special considerations?
- What changes were made to my medication during my hospital stay, and why?
- What effects can I expect from my new medications?
- What side effects should I be aware of?
- Where should I turn if I have questions or experience problems with my medications?
- Can I practice taking my medications (e.g., injections or inhalers) while in the hospital?
- What support is available if I need help with my medications at home?

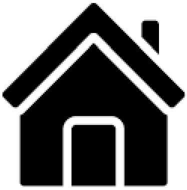

Preparing to go home

(discharge)

Discharge means it is time to leave the hospital, and there may be a lot of information for you and your relatives to discuss. This information is meant to help you understand what to expect when you return home and how to get help if needed.

Before leaving the hospital, you have the right to receive this information.

- **Discharge conversation:** A discussion with a doctor about what has happened during your hospital stay and the plan moving forward. It can be helpful if a relative is present.
- **Discharge summary with a medication report:** Written information about your hospital stay, changes made to your medications, and the next steps in your care. Read and keep the summary and show it to your relatives if needed.
- **Medication list:** A written list of the medications you should continue taking at home. Use this list and discard old ones.

Checklist before discharge

Review this checklist with healthcare staff to ensure everything is in order before leaving the hospital.

- ☐ I know which symptoms to watch for.
- ☐ I know which medications to take when I get home and how to take them.
- ☐ I know where to go for prescription renewals.
- ☐ I know where to turn if I feel worse or have questions about my medications.
- ☐ I know how home care services or home nursing will assist me with my medications (if applicable).
- ☐ I know what follow-up care is planned for me.
- ☐ I have received my discharge summary.
- ☐ I have received my updated medication list.
- ☐ I have had a discharge conversation with a doctor.

Advice for when you are home

My medications

- If you have future questions about your medications, contact a pharmacy or your healthcare provider.
- If you forget which changes were made to your medications, check the discharge summary and medication list.
- The discharge summary and medication list from the hospital are also available in your medical records at 1177.se. You can also contact your healthcare center to obtain this information again.
- Always use the latest medication list provided by the hospital or your healthcare center. The list from a pharmacy may not include all recent changes, so do not rely on it.
- Unused medications should be returned to a pharmacy.
- Remember to renew your prescriptions in time.

Seek help if you feel worse

- Call 112 or go to the nearest emergency room if you fear you are experiencing something serious or life-threatening.
- You may still feel symptoms when you first return home. Contact your healthcare center or call 1177 if the symptoms persist, worsen, or if new symptoms arise.

Notes
