# Supplementary material for: Improved Medication communication and Patient involvement At Care Transitions (IMPACT-care): study protocol for a pre–post intervention trial in older hospitalised patients
Source: BMJ Open. 2025 May 2;15(5):e099547. doi: 10.1136/bmjopen-2025-099547 (PMC12049937; doi:10.1136/bmjopen-2025-099547)
Supplement: online supplemental file 3 [file bmjopen-15-5-s003.pdf]

Complete Medication Documentation at Discharge Measure (CMDD-M)

(Unofficial English version, translated from Swedish)

Item	Discharge letter (intended for the patient)	Points
1	The discharge letter includes a description of medication changes<br>- *0 points: No*<br>- *1 point: Yes*	0-1
2	**All** medication changes are explicitly* stated *(including duration/end date if time-limited)*<br>- *0 points: No*<br>- *2 points: Yes*	0 or 2
3	Reasons for **all** medication changes are stated<br>- *2 points: The reason for **all** changes is included*<br>- *1 point: The reason for at least one change is included*<br>- *0 points: No reasons are stated*<br>o *Automatically scored 0 points if item 2 is scored 0*	0-2
**Discharge summary (intended for the next healthcare provider)**		
4	Information about medication treatment is included in the discharge summary *(sufficient if medications at discharge are listed, or if it is stated that medication changes have been made)*<br>- *0 points: No*<br>- *1 point: Yes*	0-1
5	**All** medication changes are stated<br>- *2 points: **All** medication changes are explicitly* stated*<br>- *1 point: **All** changes are stated in a general** way*<br>- *0 points: At least one change is missing, or incorrectly stated*<br>o *Automatically scored 0 points if item 4 is scored 0*	0-2
**Referral**		
6	A referral is sent to the next healthcare provider<br>- *0 points: No referral and medication changes were made*<br>- *1 point: Yes, or no referral needed (no medication changes made)*	0-1
	**Total**	0-9

* Explicitly: For initiation and changes, state the medication name, strength, dose, dosage, and dosage form. For discontinuation state the medication name.

** General: For example, "Pain relief treatment initiated".

Standard Operating Procedure (SOP) for using the CMDD-M

General Guidelines for Assessment

Identifying medication changes

- Medications at admission: Check the historical medication list from the day of admission. (Note: Admission could have been in another department.)
- Medications at discharge: Check the historical medication list from the day of discharge.
- Not considered a change:
  - Medications added or removed from the medication list during a medication reconciliation at admission (these are corrections, not changes) as noted in the doctor’s or pharmacist’s note.
  - Over-the-counter creams that can be purchased without prescription, regardless of the change made.
- Examples of how to assess combination preparations:
  - If Ramipril Comp is discontinued and Ramipril is initiated, this counts as 1 discontinuation and 1 initiation.
  - If two separate medications are switched to a combination preparation this counts as 2 discontinuations and 1 initiation.

Item-Specific Guidelines for Assessment

Items 2 and 5

- For initiation or changes to a medication, then name, strength, dose, dosage, and dosage form must be explicitly stated.
- For discontinuation, only the name must be stated.
- Medications prescribed solely for use during the hospital stay do not need to be included, such as intravenous antibiotics, insulin, infusion fluids, and similar medications.

Item 3

- The reason for a medication change may be acceptable if stated in general terms such as “for the heart”, depending on the recipient.

Item 5

- Examples of general ways to state medication changes include:
  - "Blood pressure medication reduced"
  - "Pain relief treatment initiated"
- Simply stating “new medications prescribed” is not sufficient.
