# Supplementary material for: Improved Medication communication and Patient involvement At Care Transitions (IMPACT-care): study protocol for a pre–post intervention trial in older hospitalised patients
Source: BMJ Open. 2025 May 2;15(5):e099547. doi: 10.1136/bmjopen-2025-099547 (PMC12049937; doi:10.1136/bmjopen-2025-099547)
Supplement: online supplemental file 4 [file bmjopen-15-5-s004.pdf]

Patient Involvement in Medication Communication at Hospital discharge Questionnaire (PIMCH-Q)

(Unofficial English version, translated from Swedish)

Items	I strongly disagree	I disagree	I agree	I strongly agree	Don't know
**While in hospital…**					
1. I felt involved in decisions about my medication treatment that would continue after discharge (e.g., which changes would be made).					
2. I was offered the opportunity to have an informal caregiver present during the discharge consultation.					
3. I felt involved in decisions about the follow-up of my medication treatment.					
**After returning home…**					
4. I (and/or my informal caregiver) know what changes were made to my medication treatment in the hospital (e.g., new medications, medications I should no longer use, or changes in dosage).					
5. I (and/or my informal caregiver) know why my medication treatment was changed in the hospital (e.g., due to newly discovered atrial fibrillation or high blood pressure).					
6. I feel confident that I (and/or informal caregiver) can manage my medication treatment.					
7. I (and/or informal caregiver) know where to turn if I have questions about my medication treatment.					
8. I (and/or my relative) know how my medication treatment will be followed up.					
