# Supplementary material for: Improved Medication communication and Patient involvement At Care Transitions (IMPACT-care): study protocol for a pre–post intervention trial in older hospitalised patients
Source: BMJ Open. 2025 May 2;15(5):e099547. doi: 10.1136/bmjopen-2025-099547 (PMC12049937; doi:10.1136/bmjopen-2025-099547)
Supplement: Supplementary file 5 [file bmjopen-2025-099547.reviewer_comments.pdf]

PEER REVIEW HISTORY

BMJ Open publishes all reviews undertaken for accepted manuscripts. Reviewers are asked to complete a checklist review form and are provided with free text boxes to elaborate on their assessment. These free text comments are reproduced below.

ARTICLE DETAILS

Title (Provisional)

Improved medication communication and patient involvement at care transitions (IMPACT-care): study protocol for a pre-post intervention trial in older hospitalised patients

Authors

Cam, Henrik; Franzon, Kristin; Östman, Victoria; Kälvemark Sporrong, Sofia; Kempen, Thomas Gerardus Hendrik; Nielsen, Elisabet I; Lindner, Karl-Johan; Ekelo, Beatrice; Bernsten, Cecilia; Ehlin, Ulf; Lindmark, Stina; Hadziosmanovic, Nermin; Gillespie, Ulrika

---

VERSION 1 - REVIEW

**Reviewer**	**1**
**Name**	**Flink, Maria**
**Affiliation**	**Karolinska Institutet, Neurobiology, Care Sciences and Society**
**Date**	**11-Feb-2025**
**COI**	**None**

Thank you for this well-written, interesting study protocol adressing an area in need of improvement. I have the following minor remarks:

1. Primary outcome: as I understand your intervention, it is only component 2 "Preparation of medication-related discharge documentation" that can have an effect on the primary outcome CMDD-M. Why did you choose a primary outcome that does not include the other three components?

2. Based on the statements on Page 9, line 168-172, it seems unlikely that the inclusion period for control and intervention patients both will take 6 months.

3. Training phase: How will you assess that the staff are "sufficently trained"? How will you handle new employments during the intervention period?

4. In addition to "education" it would have been interesting to have some information on the patients that may affect how they understand and apply the medication information, i.e.,

health literacy, or for example depression or fatigue. As I understand that this is not possibly to alter now, it could be interesting to include interview questions on understanding in the process evaluation.

**Reviewer**	**2**
**Name**	**Dionne, Emilie**
**Affiliation**	**Universite Laval, Sociology**
**Date**	**19-Mar-2025**
**COI**	**None**

Thank you to the author and their team for this well thought out, developed and rigorously described study protocol. This is a study that aims to implement an innovation in transition care, specifically with regards to the administration, usages and communication related to medication. The protocol propose to assess the effectiveness and satisfaction/experience of the various actors involved (carers/professionals, patients, family members) with this new procedure, and to compare it with the current practice. The protocol shows a high degree of experience and expertise by the authors and their team, with regards to the topic as well as evaluative and implementation science. Their protocol is carefully conceived, highly developed, and provide ample details and evidence that the study they design will be adequately monitored and tracked, to develop robust knowledge.

The proposed methodology a 'pre-post' intervention is adequately; the measures are robust and adequately, too.

I think this is a fine, carefully and well described, protocol, ready for publication. My sole suggestion for the authors would be that the qualitative components – including the process evaluation – would need to be integrated. While I understand that this component may be not fully 'ready', it remains an essential part of your protocol, notably given the importance the authors attribute to person-centred care. I would think, therefore, that time should be spent to integrate these aspects in this protocol, rather than 'rush' its publication (even though I understand the time sensitive aspect of publishing a protocol…). Qualitative methods – including for the purposes of a formative evaluation – have the advantage of being 'iterative', meaning that as long as the objectives are well specified, and the general methods one planned to use, it is expected that they are improved and enriched as the study progresses and knowledge is acquired regarding the 'context' and the 'object' under study. I thus would strongly advise the authors to take a little bit of time to flesh out well the qualitative components of this study as they appear particularly essential and central to the project proposed, and would also go a long way to confirm this proposed study as a truly person-centred project.

Additionally, if possible, please indicate if there is an advisory board composed of people with lived or practical experience; or if such individuals are members of the research team; if not, please indicate why there is no lay member involved in the conception of the study.

Hence, to sub up:

— please provide more information about the qualitative components of this study

— please provide more information regarding the research team and any advisory committee composed of non-researcher members, specifically patient partners, family members, citizen partners.

**** **The reviewer provided a marked copy with additional comments. Please contact the publisher for full details.** ****

VERSION 1 - AUTHOR RESPONSE

Responses to the comments of Reviewer #1

Thank you for this well-written, interesting study protocol adressing an area in need of improvement.

Response: Thanks for the positive feedback on our manuscript.

I have the following minor remarks:

1. Primary outcome: as I understand your intervention, it is only component 2 "Preparation of medication-related discharge documentation" that can have an effect on the primary outcome CMDD-M. Why did you choose a primary outcome that does not include the other three components?

Response: We understand the reviewer's concern. The choice of primary outcome was carefully considered and discussed extensively within the research team. We have added a paragraph to explain our reasons for choosing the primary outcome, see Page 20, line 445-458.

2. Based on the statements on Page 9, line 168-172, it seems unlikely that the inclusion period for control and intervention patients both will take 6 months.

Response: We anticipate that both the control and intervention periods will require approximately six months of active patient inclusion. The intervention will pause during the summer due to staff vacations. We expect to include patients at a similar pace during both periods. However, we included this paragraph as a precaution in case unforeseen factors, such as increased workload or staff turnover affecting the pharmacists delivering the intervention.

3. Training phase: How will you assess that the staff are "sufficently trained"? How will you handle new employments during the intervention period?

Response: We agree that we cannot fully assess whether the healthcare professionals are "sufficiently" trained. The wording in page 5, line 120-121 is amended to: "Once the HCPs have undergone training sessions…". However, we plan to schedule regular meetings with the pharmacists delivering the intervention, during which barriers such as insufficient training of HCPs may be identified and addressed. We have added a paragraph under the subheading Qualitative process evaluation Page 23, line 543-545 to reflect this.

Regarding newly employed staff, we have clarified the wording on Page 11, line 233-234 to address this.

4. In addition to "education" it would have been interesting to have some information on the patients that may affect how they understand and apply the medication information, i.e., health literacy, or for example depression or fatigue. As I understand that this is not possibly to alter now, it could be interesting to include interview questions on understanding in the process evaluation.

Response: Thanks for the suggestion. We will consider it in the planning of the qualitative process evaluation (please see our response to comment 1 from reviewer #2 regarding this evaluation).

Responses to the comments of Reviewer #2
Thank you to the author and their team for this well thought out, developed and rigorously described study protocol. This is a study that aims to implement an innovation in transition care, specifically with regards to the administration, usages and communication related to medication. The protocol propose to assess the effectiveness and satisfaction/experience of the various actors involved (carers/professionals, patients, family members) with this new procedure, and to compare it with the current practice. The protocol shows a high degree of experience and expertise by the authors and their team, with regards to the topic as well as evaluative and implementation science. Their protocol is carefully conceived, highly developed, and provide ample details and evidence that the study they design will be adequately monitored and tracked, to develop robust knowledge.
The proposed methodology a 'pre-post' intervention is adequately; the measures are robust and adequately, too.
I think this is a fine, carefully and well described, protocol, ready for publication.

Response: Thanks to the reviewer for the positive feedback on our manuscript.

1. My sole suggestion for the authors would be that the qualitative components – including the process evaluation – would need to be integrated. While I understand that this component may be not fully 'ready', it remains an essential part of your protocol, notably given the importance the authors attribute to person-centred care. I would think, therefore, that time should be spent to integrate these aspects in this protocol, rather than 'rush' its publication (even though I understand the time sensitive aspect of publishing a protocol…). Qualitative methods – including for the purposes of a formative evaluation – have the advantage of being 'iterative', meaning that as long as the objectives are well specified, and

the general methods one planned to use, it is expected that they are improved and enriched as the study progresses and knowledge is acquired regarding the 'context' and the 'object' under study. I thus would strongly advise the authors to take a little bit of time to flesh out well the qualitative components of this study as they appear particularly essential and central to the project proposed, and would also go a long way to confirm this proposed study as a truly person-centred project.
Hence, to sub up:
— please provide more information about the qualitative components of this study

Response: We have included more information about the planned qualitative process evaluation. Please see under that heading on page 24 line 543-561.

2. Additionally, if possible, please indicate if there is an advisory board composed of people with lived or practical experience; or if such individuals are members of the research team; if not, please indicate why there is no lay member involved in the conception of the study.
— please provide more information regarding the research team and any advisory committee composed of non-researcher members, specifically patient partners, family members, citizen partners.

Response: We expanded the information about the research team on page 9 line 184 - 192, under the subheading "Intervention development".
Details about the advisory board and whether any of its members are part of the research team were already included under the subheading *"Public and patient involvement"* on page 28, lines 652–663. We have refined the wording in this section to improve clarity.
